# Identification and validation of N6-methyladenosine (m6A)-related lncRNAs signature for predicting the prognosis of laryngeal carcinoma, especially for smoking patients

**DOI:** 10.3389/fgene.2023.1292164

**Published:** 2023-11-08

**Authors:** Yuqing Chen, Chenyu Chen, Gufeng Gao, Chaojun Zeng, Zhifeng Chen, Gongbiao Lin, Guangnan Yao, Shenqing Nian, Xihang Chen, Simin Weng, Xi Gu, Chang Lin

**Affiliations:** ^1^ Department of Otorhinolaryngology, Head and Neck Surgery, The First Affiliated Hospital of Fujian Medical University, Fuzhou, Fujian, China; ^2^ Department of Otorhinolaryngology, Head and Neck Surgery, National Regional Medical Center, Binhai Campus of the First Affiliated Hospital of Fujian Medical University, Fuzhou, Fujian, China; ^3^ Fujian Provincial Clinical Medical Research Center for Ear, Nose and Throat Difficulty Diseases, Fuzhou, Fujian, China; ^4^ Department of Geriatrics, The First Affiliated Hospital of Fujian Medical University, Fuzhou, Fujian, China

**Keywords:** laryngeal squamous cell carcinoma, long non-coding RNAs, N6-methyladenosine, smoking history, drug sensitivity

## Abstract

Laryngeal cancer (LC), a highly fatal tumor in the head and neck region, has been the focus of research in recent years. The study of LC has primarily focused on the role of long non-coding RNAs (lncRNAs) in regulating gene expression, as they have emerged as pivotal factors in this biological process. Additionally, a reversible RNA modification called N6-methyladenosine (m6A) has been observed to have a significant impact on gene expression as well. The purpose of this research is to investigate the impact of m6A-related lncRNAs on the prognosis of laryngeal squamous cell carcinoma (LSCC). Specifically, this investigation analyzed the m6A-related regulators’ patterns of expression and mutation, encompassing a total of 15 regulators. Drawing upon the expression levels of prognostic m6A-regulated lncRNAs, two distinct lncRNA clusters were identified. Further analysis revealed differentially expressed lncRNAs between these clusters. In addition to studying the expression of lncRNAs, the researchers also examined the distribution of clinical characteristics and the tumor microenvironment (TME) in relation to the identified lncRNA clusters. This provided valuable insights into potential associations between lncRNA expression patterns and the clinical features of LSCC. Through the establishment of a risk model associated with lncRNAs, we were able to further investigate their clinical features, prognosis, and immune status. Additionally, we conducted a separate analysis of LINC00528, a lncRNA associated with smoking, examining its expression, overall survival time, correlated mRNAs, and conducting enrichment of Gene Ontology (GO) and Kyoto Encyclopedia of Genes and Genomes (KEGG), as well as determining the sensitivity of related drugs. RT-qPCR results also indicated an increase in LINC00528 expression among smoking LSCC patients. The findings suggest that a high expression level of LINC00528 in LSCC patients may lead to a more favorable prognosis, providing new insights for the management and treatment of LSCC patients, particularly those with high expression of LINC00528. Overall, this research sheds light on the prognostic impact of m6A-regulated lncRNAs in LSCC. The implications of these findings for the advancement of innovative therapeutic approaches for LSCC patients are noteworthy.

## 1 Introduction

Laryngeal carcinoma (LC), a prevalent form of head and neck cancer, causing numerous fatalities worldwide, added over 180,000 new cases and 99,000 dead cases worldwide in 2020 (Yan et al., 2022). The most prevalent form of LC is laryngeal squamous cell carcinoma (LSCC) (Luo et al., 2022), which is typically treated with radical surgery or radiation therapy in its early stages (Pfister et al., 2006; Du et al., 2020). The LSCC patients’ 5-year overall survival (OS) at early stage treated by transoral laser surgery or radiation therapy was about 87%–92% (Pakkanen et al., 2022). However, the survival rate for locally advanced LSCC patients is considerably lower, just about 39%–55% (Megwalu and Sikora, 2014; Steuer et al., 2017), and the occurrence of metastases and relapses significantly impacts the prognosis. Therefore, it is crucial to identify biomarkers that can facilitate early diagnosis and improve patient outcomes.

The regulation of gene expression involves a critical function of RNA modification, with a specific focus on N6-methyladenosine (m6A) modification (He, 2010; Sun et al., 2019). Various human diseases, including hypertension (Paramasivam et al., 2020), cardiac hypertrophy (Dorn et al., 2019), and cancer (Li et al., 2017; Sun et al., 2019), have been associated with m6A RNA methylation. Previous research has demonstrated the involvement of m6A methylation in LSCC progression (Wang et al., 2021), making it a potential target for therapeutic intervention (Gu et al., 2020).

Long non-coding RNAs (lncRNAs), RNA molecules exceeding 200 nucleotides in length (Ho et al., 2021; Pisignano and Ladomery, 2021; Taniue and Akimitsu, 2021), play a significant role in the regulation of gene expression as they do not encode proteins (Taniue and Akimitsu, 2021; Winkle et al., 2021). Researchers have confirmed that lncRNAs serve as promising biomarkers and therapeutic targets for LSCC (Lv et al., 2022). For instance, the downregulation of lncRNA HCP5/miR-216a-5p/ZEB1 axis has been shown to inhibit the malignant behavior of LSCC cells (Zhang et al., 2022).

Additionally, m6A-related lncRNAs have been recognized for their role in cancer diagnosis, prognosis, and treatment (Li et al., 2021; Lv et al., 2021; Weng et al., 2021; Liu et al., 2022a). However, the precise function of m6A methylation-associated lncRNAs in LSCC remains unclear. Further research is needed to fully understand their impact and potential applications in improving patient care. Hence, it is crucial to investigate the lncRNAs linked to m6A methylation in LSCC and ascertain potential prognostic biomarkers. Additional research is imperative.

In the present investigation, we investigated the crucial lncRNAs associated with m6A in laryngeal samples, comparing individuals with LSCC to healthy donors. We quantified the expression of each lncRNA in every LSCC patient and conducted network analysis to investigate the relationship between m6A and lncRNAs. We categorized all differentially expressed m6A-related lncRNAs into two clusters based on significant prognostic disparities. Furthermore, we determined the immune cells related risk, which could serve as potential biomarkers for immunotherapy and therapeutic targets for LSCC. Subsequently, we assessed the distinct impacts of each clinical phenotype on prognosis, depicting the respective survival curves. Finally, we identified a differentially expressed m6A-related lncRNA and immune cell in smoking and non-smoking LSCC patients. We separately analyzed the expression, overall survival time, associated mRNAs, and conducted Gene Ontology (GO), Kyoto Encyclopedia of Genes and Genomes (KEGG), and sensitivity analysis of relevant drugs for LINC00528. This investigation could potentially unveil key factors for predicting survival prognosis and identifying novel treatment targets for LSCC individuals.

## 2 Materials and methods

### 2.1 Dataset source

To ensure a rigorous analysis and minimize any statistical bias, exclusively LSCC samples from The Cancer Genome Atlas (TCGA) database (https://portal.gdc.cancer.gov) were included in our study. We retrieved a comprehensive collection of 123 transcriptome files along with their corresponding clinical data, focusing solely on samples that presented complete survival and follow-up information. The analysis focused on 23 m6A-related genes, which included writers (METTL3, METTL14, METTL16, WTAP, VIRMA, ZC3H13, RBM15, RBM15B), readers (YTHDC1, YTHDC2, YTHDF1, YTHDF2, YTHDF3, HNRNPC, FMR1, LRPPRC, HNRNPA2B1, IGFBP1, IGFBP2, IGFBP3, RBMX), and erasers (FTO, ALKBH5).

### 2.2 Identification of m6A-related lncRNAs in LSCC

R software package of “limma” was employed to extract lncRNAs exhibiting differential expression, while the connection between these lncRNAs and m6A modulators was examined via Spearman correlation analysis. To identify m6A-associated lncRNAs, the criteria for screening were set as follows: coefficient > 0.4, *p* < 0.001. The accuracy of the outcomes was confirmed by generating network diagrams with the assistance of the “igraph” package in the R programming language.

### 2.3 Consensus clustering of m6A-related lncRNAs

Based on the levels of lncRNAs expression associated to m6A modification, patients diagnosed with LSCC were categorized into two groups (cluster 1 and cluster 2). The classification was carried out through the utilization of cumulative distribution function (CDF) and optimal k-means clustering techniques. The software package “ConsensusClusterPlus” in R was employed to conduct the cluster analysis, while the R packages “survival” and “survminer” were utilized to calculate the OS data for each cluster using the Kaplan-Meier method. The impact of m6A-related lncRNAs on clinical characteristics was examined based on information extracted from the TCGA database. For the estimation of the tumor immune microenvironment, the ESTIMATE algorithm was employed.

### 2.4 Clustering of m6A-associated lncRNAs based on consensus

The development of the risk score model in LSCC is established. Initially, all lncRNAs from the network underwent individual analysis using univariate Cox regression (*p* < 0.05). Subsequently, the least absolute shrinkage and selection operator (LASSO)-Cox regression method was utilized to further explore prognostic lncRNAs associated with m6A. The risk score equation for individual m6A-related lncRNA was formulated as follow:

$$Risk\;score=\sum_{1}^{n}x_{i}*y_{i}$$

incorporating x_i_ as the expression level of each lncRNA and y_i_ as its corresponding coefficient. Using the risk score median as a basis, the patients diagnosed with LSCC were grouped them into two categories indicating high- and low-risks. Prognostic modeling was conducted employing multivariate Cox regression analysis. To visualize the outcomes, forest plots were utilized.

### 2.5 Examination of survival rates and assessment of the risk score model

We utilized R package of “survival” to conduct survival analysis on the identified lncRNAs, employing Kaplan-Meier curves. To illustrate the survival status and time, as well as risk levels of both the low-risk and high-risk groups, a scatterplot was generated. We randomly assigned 123 patients into two equal-sized groups, training and testing, following a 1:1 ratio, for validating the risk score model. Assessing the precision of the model in forecasting patient survival, we utilized the R package “timeROC” to generate receiver operating characteristic (ROC) curves and determined its accuracy by evaluating the area under curve (AUC).

### 2.6 Evaluation of independence and stratification analysis pertaining to the established model

The heatmap in the form of a R package called “pheatmap” illustrated the expression profiles and clinical characteristics of key m6A-related lncRNAs in both low- and high-risk groups. Additionally, in order to assess the risk score of LSCC individuals, we consecutively conducted univariate and multivariate Cox regression analyses and incorporated age, gender, TNM stage, grade, and risk score as variables independent of one another in the Cox regression analysis. Utilizing the findings from the Cox multiple regression analysis, we conducted a stratified analysis to delve deeper into whether the risk model functions independently as a prognostic factor for this particular group of LSCC patients.

### 2.7 Formulation and verification of the nomogram

To assess the survival probabilities of patients with LSCC at 1-, 3-, and 5-year intervals, we employed a multivariate Cox regression analysis. This analysis was instrumental in identifying several independent prognostic factors. Consequently, a prognostic nomogram was devised to aid in predicting the outcome. To undertake these analyses, we utilized the “rms” R package, which facilitated the evaluation of survival probabilities for LSCC patients.

### 2.8 Estimations of immune infiltration in relation to the risk score model

The estimation resource for tumor immunity was acquired from TIMER (http://timer.cistrome.org/). TIMER, CIBERSORT, quanTIseq, xCell, MCP-counter, and EPIC algorithms were employed to analyze the estimations of immune infiltration. We employed the R software package “limma” to demonstrate the correlations among the risk score, immune functions, and the expression of checkpoint inhibitors.

### 2.9 Functional and enrichment analyses

The analysis of gene functions and genomic information was conducted using GO analysis and KEGG (adjusted *p*-value < 0.05, |logFC| > 1). To assess gene annotation and gene product analysis, GO analysis encompasses biological process (BP), molecular function (MF), and cellular component (CC) (Ashburner et al., 2000). Additionally, KEGG, a database resource, provides higher-order functional information for comprehensively understanding gene functions and genomic analyses (Kanehisa and Goto, 2000). The functional and enrichment analyses were carried out utilizing various R packages, namely, “clusterProfiler,” “org.Hs.eg.db,” “enrichplot,” “ggplot2,” “RColorBrewer,” “dplyr,” and “ComplexHeatmap.” These packages were instrumental in facilitating the analysis and demonstration of functional and enrichment outcomes.

### 2.10 Drugs sensitivity prediction

Using the R package “oncoPredict,” the prediction of drug susceptibility for LINC00528 was conducted. To estimate drug sensitivity, AUC was measured and compared across different levels of LINC00528 expression. A higher sensitivity corresponds to a lower AUC value.

### 2.11 Correcting and processing steps of LSCC tissues

Tumor tissues and adjacent non-tumor tissues were collected from seven patients who received a diagnosis of LSCC at the First Affiliated Hospital of Fujian Medical University. The sample group consisted of three non-smokers and four smokers. Prior to surgery, all patients underwent either partial or total laryngectomy, without receiving any form of chemotherapy or radiotherapy. Prior to enrollment in the study, written informed consent was acquired from all patients (or their guardians).

### 2.12 Extraction of RNA and quantitative real-time PCR

To extract RNA from LSCC tissues, the Fast-Pure Cell/Tissue Total RNA Isolation KIT V2 (Vazyme, Nanjing, China) was utilized. For cDNA synthesis, the HiScript II Q RT SuperMix for qPCR (+gDNA wiper) (Vazyme, Nanjing, China) was chosen. Subsequently, Quantitative RT-PCR was conducted using the HRbioTM qPCR SYBR Green Master Mix (No Rox) (Herui, Fuzhou, China) on the LightCycler^®^ 96 System (Roche Diagnostics, Mannheim, Germany). The program for amplification comprised a primary denaturation procedure at a temperature of 95°C for a duration of 5 min, followed by a total of 40 cycles at 95°C for 10 s and 60°C for 30 s. To determine the relative expression level of lncRNA, the 2^(−∆∆Ct) method was employed. The primer pair (5′ to 3′) used for Quantitative RT-PCR was as follows: LINC00528: F: ATA​GTC​TGG​GAT​GGT​CAT​TTT​CGG, and R: GCT​GCA​GTC​CCA​GCA​TTA​TCT​GTA; GAPDH: F: GGT​GTG​AAC​CAT​GAG​AAG​TAT​GA, and R: GAG​TCC​TTC​CAC​GAT​ACC​AAA​G.

### 2.13 Statistical analysis

Perl and R software (version 4.2.1) were utilized for performing the statistical analysis and generating the figures, along with RStudio (2022.07.1+554). A *p*-value < 0.05 was considered statistically significant, with two-sided statistical tests. **** indicates *p*-value < 0.0001; *** indicates *p*-value < 0.001; ** indicates *p*-value < 0.01; * indicates *p*-value < 0.05, and ns indicates no significance.

## 3 Results

### 3.1 Identification of lncRNAs associated with m6A modification in LSCC

**FIGURE 1:**
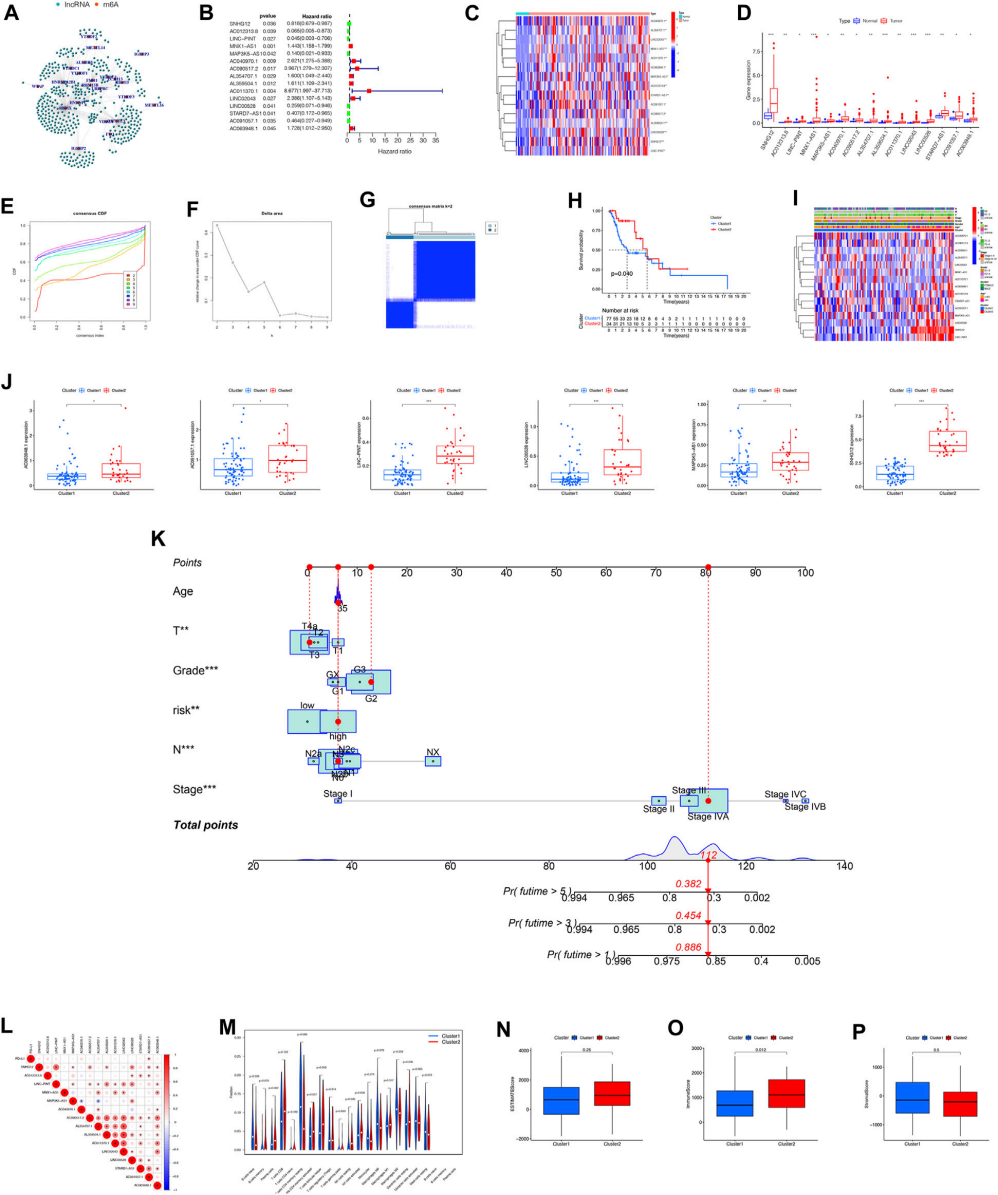
Variations in the expression and unsupervised clustering analysis of lncRNAs associated with m6A modification. **(A)** Summary graphs illustrating the expression patterns of m6A-related lncRNAs. **(B)** Prognostic significance of m6A-related lncRNAs determined by univariate Cox regression. **(C)** Comparative analysis of m6A-related lncRNAs in normal and tumor samples. Heatmap displaying the discrepancies in expression levels between normal and tumor samples. **(D)** Expression patterns of m6A-related lncRNAs identified through univariate Cox regression analysis. **(E)** CDF curve for unsupervised clustering analysis of consensus. **(F)** Delta area under CDF curve for cluster analysis. **(G)** Identification of two distinct gene clusters based on DEGs of m6A clusters 1 and 2. Identification of two distinct gene clusters utilizing unsupervised consensus clustering analysis, with the inclusion of 15 prognostic DEGs from m6A clusters 1 and 2. **(H)** Analysis of OS differences between m6A clusters 1 and 2 using Kaplan-Meier survival analysis. Log-rank *p*-value = 0.04. **(I)** Heatmap illustrating the variations in m6A-related lncRNAs and clinical features between the two clusters. **(J)** Differential expression levels of m6A-related lncRNAs between cluster 1 and cluster 2. **(K)** Prognostic nomogram for predicting the survival of LSCC patients based on the TCGA cohort. **(L)** Pearson correlation analysis conducted to determine the correlations among m6A RNA methylation regulators. **(M)** Tumor immune dysfunction and exclusion analysis performed to predict the sensitivity of patients in high- and low-risk groups to immunotherapy for tumor immune dysfunction and exclusion. **(N–P)** Comparison of immune score composition, stromal score composition, and estimate score composition between cluster 1 and cluster 2. Abbreviation: CDF, Cumulative Distribution Function; OS, Overall Survival; DEGs, Different Expression Gene.

Out of 417 lncRNAs, 23 m6A regulators showed significant associations, while the rest had only minimal correlation with lncRNAs. The network graph displayed in Figure 1A represents the interconnections among these lncRNAs and m6A regulators. To pinpoint lncRNAs with prognostic significance, univariate Cox regression analysis was conducted. Notably, 15 lncRNAs (SNHG12, AC012313.8, LINC−PINT, MNX1−AS1, MAP3K5−AS1, AC040970.1, AC090517.2, AL354707.1, AL359504.1, AC011370.1, LINC02043, LINC00528, STARD7−AS1, AC091057.1, AC063948.1) within the TCGA-LSCC cohort exhibited significant correlations with the survival rates of LSCC patients (Figures 1B–D).

### 3.2 Characterization of m6A clusters and correlation with clinical traits using m6A-related lncRNAs

Our research analyzed the impact of m6A-regulated lncRNAs on the development and outlook of LSCC patients. To achieve this, the study conducted unsupervised analysis through the utilization of the Consensus Cluster Plus (CCP) approach. The expression matrix of 15 prognostic lncRNAs was utilized for this analysis. Based on the consensus matrix (Figures 1E–G), we categorized the patients into two distinct clusters: cluster 1, comprising 77 cases, and cluster 2, consisting of 34 cases. We determined the optimal clustering parameter as k = 2. By examining the Kaplan-Meier survival curve, we observed a notable divergence in survival rates between the two clusters (Figure 1H). To further investigate the variation in levels of lncRNA expression and clinical characteristics between the two clusters, we conducted a heatmap analysis (Figure 1I). The boxplot analysis revealed notable distinctions in the levels of expression for 6 m6A-associated lncRNAs (AC063948.1, AC091057.1, LINC-PINT, LINC00528, MAP3K5-AS1, SNHG12) within the two clusters between the two clusters (Figure 1J). In order to develop a prognostic tool that can be used in clinical settings to forecast the outlook of patients with LSCC, we constructed a prognostic nomogram utilizing the TCGA cohort. The nomogram allows for the prediction of survival probabilities at 1-, 3-, and 5-years. The prediction model incorporates five distinct prognostic factors: T stage, N stage, Stages, Gender, and risk score. These variables were included in the model as independent predictors (Figure 1K). The coefficients of each factor were shown in the Supplementary Table S1.

### 3.3 The immune score of each cluster in LSCC

Examining the potential impact of tumor microenvironment (TME) on patient survival through immune infiltration regulation, our focus shifted towards investigating the potential involvement of immune-related factors in driving disparate clinical outcomes observed between the two clusters (Figures 1L, M). Applauded for its accuracy, the ESTIMATE algorithm analysis played a crucial role in assessing the precise estimate score, immune score, and stromal score within the two clusters. Remarkably, our findings indicated a lower immune score in cluster 1, contrasting it with cluster 2. Conversely, the estimate score and stromal score exhibited no statistically significant differences between the two clusters (Figures 1N–P).

### 3.4 Development and confirmation of a risk score model

**FIGURE 2:**
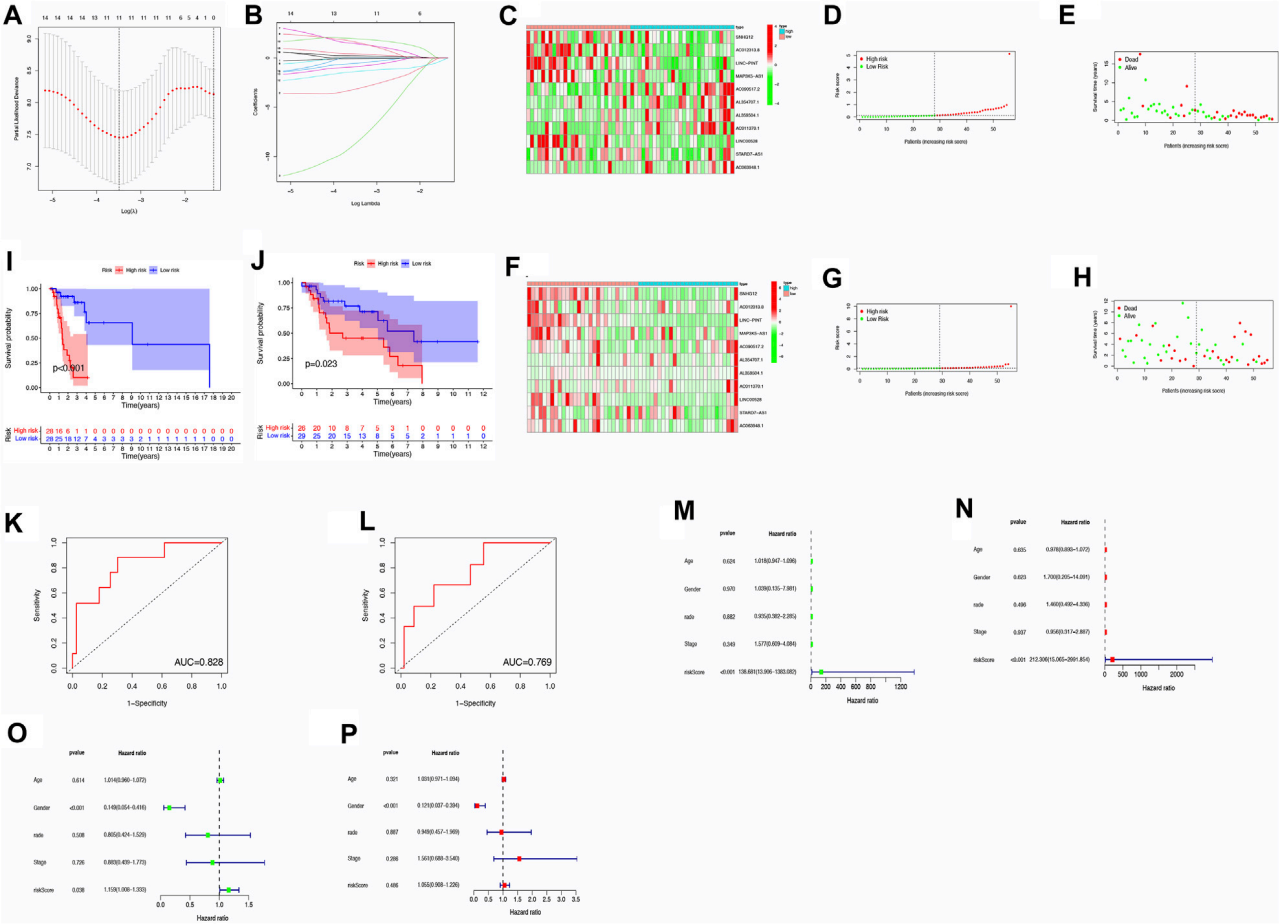
Analysis of risk scores through correlation. **(A)** Tuning parameter selection in LASSO regression through cross-validation. **(B)** LASSO regression involving 9 m6A-related prognostic lncRNAs. **(C)** Heatmap displaying differences in m6A-related lncRNAs between low- and high-risk groups within the train group. **(D)** Distribution of risk scores within the training group. **(E)** Distribution of survival status within the training group. **(F)** Heatmap illustrating differences in m6A-related lncRNAs between low- and high-risk groups within the test group. **(G)** Distribution of risk scores within the testing group. **(H)** Distribution of survival status within the testing group. **(I–L)** OS analysis for high/low risk patients and ROC curve measuring predictive value in the training cohort **(I,K)** and test cohort **(J,L)**. **(M–P)** Assessment of the independence and effectiveness of this prognostic model for LSCC patients. Forest plots depicting univariate **(M,O)** and multivariate **(N,P)** Cox regression analyses in LSCC within the train group **(M,N)** and test group **(O,P)**. Abbreviation: OS, Overall Survival; ROC, receiver operating characteristic.

Through the utilization of the risk score model, it was determined that this particular prognostic tool holds significant efficacy in forecasting survival outcomes for patients with LSCC. The model was able to effectively categorize patients into various risk groups according to their individual survival status. Notably, the high-risk group had a significantly higher number of patients who had unfortunately passed away in comparison to the low-risk group. This was demonstrated in Figures 2A–H, where the risk plot clearly showed the separation of patients into distinct risk groups. Furthermore, the Kaplan-Meier survival curve, as shown in Figures 2I, J, indicated that patients categorized under the low-risk cohort demonstrated a better OS when contrasted with individuals in the high-risk group (*p* < 0.05). To further investigate the predictive ability of the risk model in determining survival, an analysis of the ROC curve was performed. The efficacy of a prognostic model is evaluated using the ROC curve, wherein the AUC serves as a measure showcasing its precision. In this study, the AUC values for the prognostic risk score were 0.769 in the test group and 0.828 in the train group (Figures 2K, L). The findings presented herein indicate that the model for risk scoring exhibits a commendable capacity in forecasting the survival results among patients diagnosed with LSCC.

### 3.5 Evaluation of the risk score model as a standalone prognostic tool and for stratified analysis

To evaluate the independent predictive ability of the risk scoring system, univariate and multivariate Cox regression analyses were performed. The main objective was to ascertain if prognosis could be accurately predicted. The clinical features included in the analysis were age (≤65 years vs. >65 years), gender (female vs. male), grade (I–II vs. III–IV), T stage (1–2 vs. 3–4), N stage (0 vs. 1–3), M stage (0 vs. 1) and TNM stage (I–II vs. III–IV) (Figures 2M–P).

### 3.6 Correlations between the risk model and immune infiltration

**FIGURE 3:**
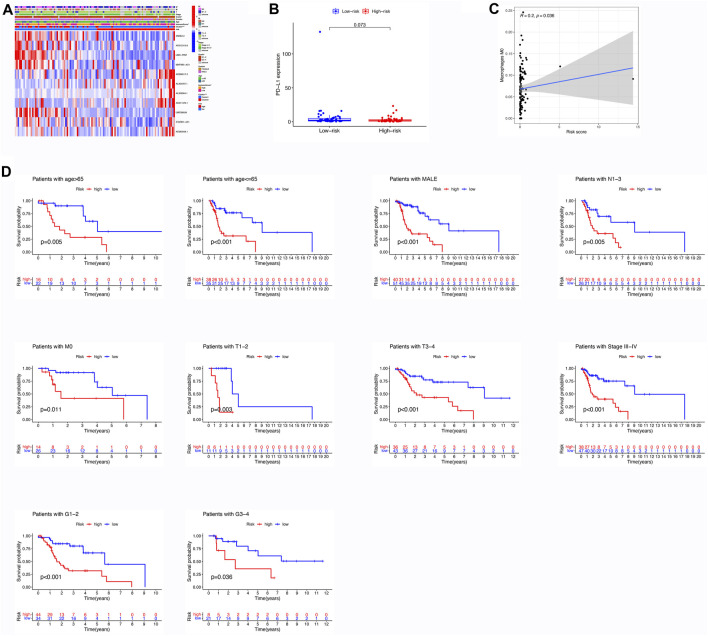
Assessment of Differential Expression Levels, Kaplan-Meier Analysis, and Immune Cell Infiltration based on Risk Score. **(A)** Heatmap illustrating the variances in m6A-related lncRNAs and clinical features between the low- and high-risk groups. **(B)** Comparison of expression levels between the high- and low-risk groups in PD-L1. **(C)** Correlation between the risk score and immune cell infiltration; specifically, a positive correlation exists between the risk score and the abundance of M0 macrophages. **(D)** Evaluation of the prognostic value of the m6A-associated lncRNA signature in patients diagnosed with LSCC. Kaplan-Meier analysis was performed to classify different risk groups based on clinical factors, including age, gender, M stage, N stage, T stage, grade, and TNM stage.

**FIGURE 4:**
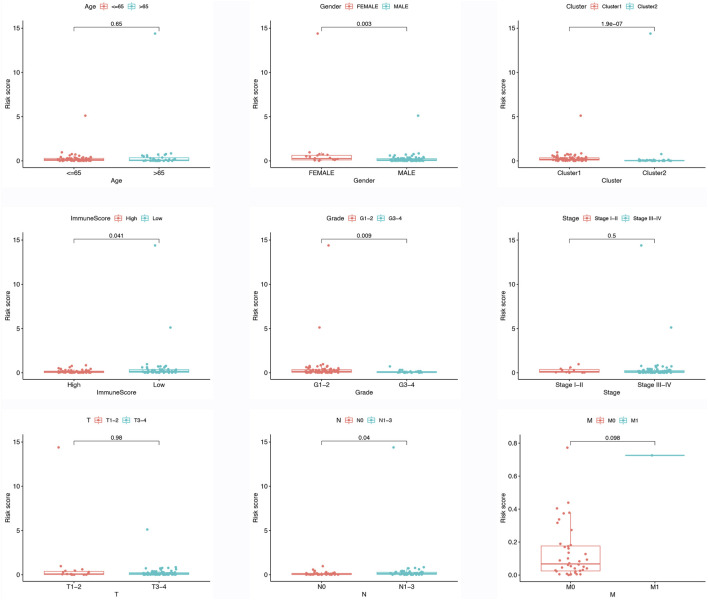
Different risk score in different clinical features, include age, gender, cluster, immune score, grade, TNM stage, T stage, N stage, M stage.

Figure 3A illustrates the contrasting expression patterns of 15 lncRNAs associated with m6A in the cohorts categorized as high-risk and low-risk. The disparity in PD-L1 expression, which serves as an indicator of immune response, was observed between these risk groups (Figure 3B). Moreover, a positive correlation was observed between the risk score and the infiltration of M0 macrophages, suggesting that as the score escalates, there is an enhanced extent of macrophage invasion within the tumor (Figure 3C). An investigation was conducted to explore the correlation between the risk scores and diverse clinical characteristics, including age, gender, TNM stage, T, N, M, grade, cluster, and immune score. Interestingly, it was found that the distribution of cluster, grade, N, and immune score differed between the low- and high-risk groups (Figure 3D). Patients with male gender, cluster 2, N0, grade 3–4, and high immune scores had a higher probability of acquiring reduced risk scores (Figure 4).

### 3.7 Correlations and immune infiltration between smoking and non-smoking LSCC patients

**FIGURE 5:**
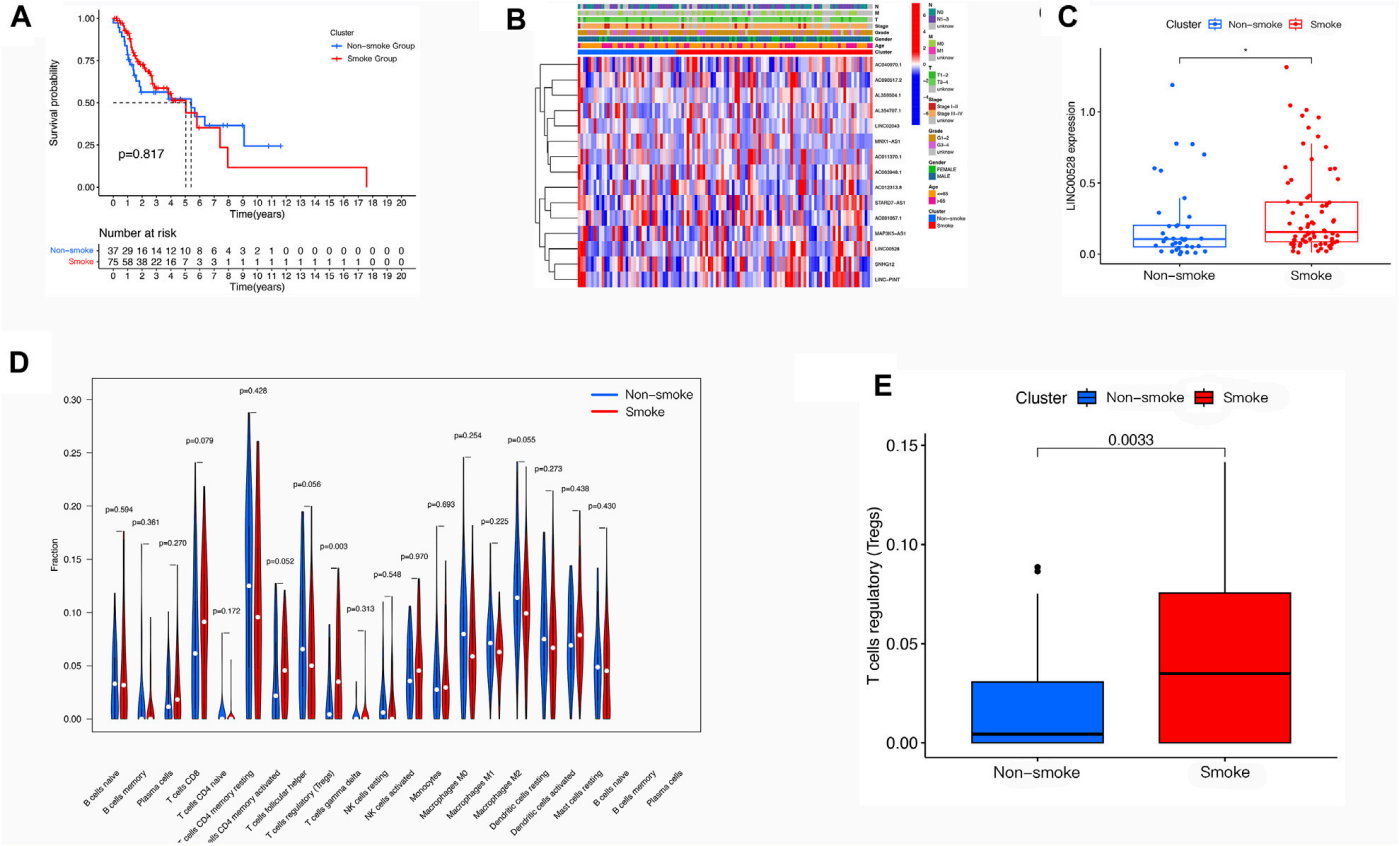
Evaluation of Smoke-Related Correlations in LSCC. **(A)** Assessment of the m6A-related lncRNA signature in LSCC patients, categorized by smoking status. **(B)** Heatmap illustrating the varying expression profiles of m6A-related lncRNAs and discrepancies in clinical characteristics between LSCC patients who smoke and those who do not smoke. **(C)** Comparative analysis of the expression levels of m6A-related lncRNAs in LSCC patients, based on smoking status. **(D,E)** Utilization of tumor immune dysfunction and exclusion analysis to forecast the response to immunotherapy in LSCC patients who smoke and those who do not smoke.

The analysis of survival probability as determined by Kaplan-Meier revealed no notable difference between the group that smoked and the group that did not smoke (Figure 5A). Additionally, smoking did not impact any of the clinical characteristics, as indicated by the heatmap (Figure 5B). However, LINC00528 displayed differential expression when comparing the two groups (Figure 5C). Subsequently, we conducted a thorough examination of immune infiltration in patients who smoke. Our findings indicate a significant increase in regulatory T cells within the smoking group (Figures 5D, E).

### 3.8 LINC00528 might lead to longer OS time in LSCC patients

**FIGURE 6:**
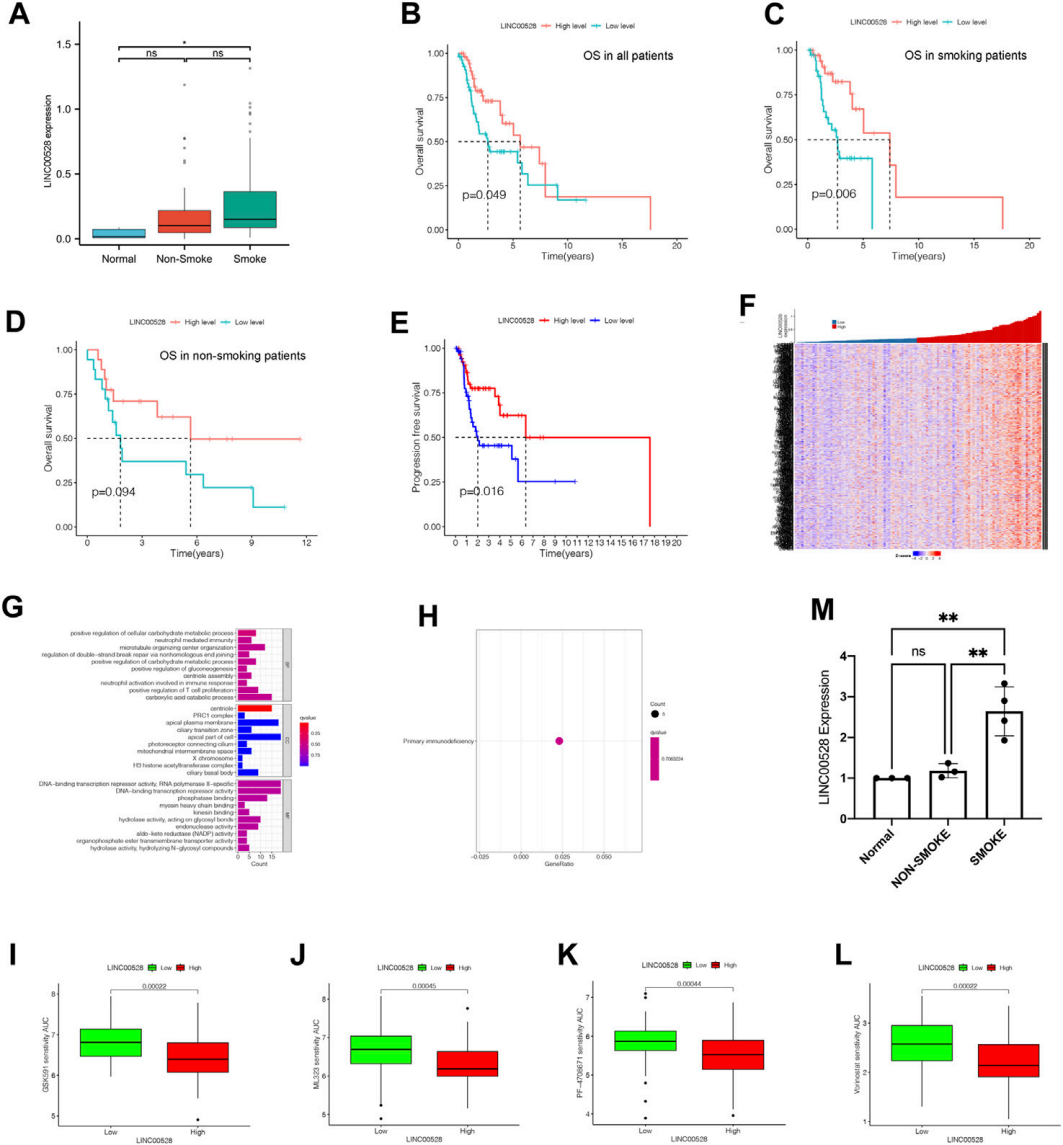
Specific analysis of LINC00528 in relation to LSCC. **(A)** Comparison of expression levels of LINC00528 in normal, non-smoke group and smoke group. **(B–D)** Overall survival analysis for patients with high and low expression levels of LINC00528: in all patients **(B)**, in smoke patients **(C)**, in non-smoke patients **(D)**. **(E)** Progression-free survival analysis for patients with high and low expression levels of LINC00528. **(F)** A heatmap depicting the differential expression of LINC00528-related mRNAs based on LINC00528 expression levels. **(G)** BP, CC, and MF associated with LINC00528-related mRNAs. **(H)** KEGG analysis indicating pathway enrichment of LINC00528-related mRNAs. **(I–L)** Prediction of drug sensitivity in LSCC patients with high and low expression levels of LINC00528, using AUCs generated by OncoPredict. Lower AUC values indicate higher sensitivities. **(M)** LINC00528 expression calculated by the result of RT-qPCR in normal, non-smoke and smoke group. Abbreviation: AUC, Area Under Curve.

In contrast with normal group, smoke group showed statistically significant increase in expression of LINC00528, while the expression of LINC00528 in non-smoke group was not notable increased compared to normal group (Figure 6A). The analysis of Kaplan-Meier demonstrated that individuals exhibiting elevated levels of LINC00528 showcased enhanced overall survival rates in contrast to those individuals displaying lower expression levels. This result was consistent in all patients with LSCC (Figure 6B) as well as in those with a smoking history (Figure 6C). No statistical difference could be observed in the expression of LINC00528 among non-smoking patients (Figure 6D). Moreover, a positive correlation was found between elevated levels of LINC00528 and increased rates of PFS (Figure 6E). Further analysis of the possible related mRNA of LINC00528 revealed their expression patterns in a heatmap (Figure 6F, Supplementary Table S2) and their functional annotations in terms of BP, CC, and MF (Figure 6G). The KEGG enrichment analysis indicated that the mRNA related to LINC00528 were significantly concentrated in the pathway of primary immunodeficiency (Figure 6H). Utilizing the OncoPredict package, different drugs were identified as potential treatment options for LSCC patients with high expression of LINC00528 based on their sensitivity scores and IC50 values (Chen et al., 2022). Among these candidate drugs were GSK591, ML323, PF-4708671, and Vorinostat (Figures 6I–L). To confirm the findings from the TCGA dataset analysis, RT-qPCR was performed on tumor tissues and adjacent normal tissues from 7 LSCC patients with different smoking histories (Figure 6M). The expression of LINC00528 was found to be significantly higher in tumor tissues from individuals with a smoking history when compared to non-smoking tissues or adjacent normal tissues. This suggests that smoking may have a role in upregulating the expression of LINC00528 in tumor tissues. Nevertheless, there existed no notable statistical disparity in the expression level of LINC00528 between malignant tissues devoid of any smoking background and neighboring healthy tissues. These experimental findings validated the differential expression of LINC00528 in the different groups calculated in the TCGA database.

## 4 Discussion

Recent studies have demonstrated the importance of m6A RNA methylation in various human diseases such as cancers (Liu et al., 2022b), hypertension (Paramasivam et al., 2020), diabetes (Yang et al., 2019), and viral infections (Williams et al., 2019). An investigation conducted specifically centered on the ALKBH5 m6A demethylase and its involvement in regulating the expression of KCNQ1OT1, consequently influencing the growth, infiltration, and spread of LSCC (Li et al., 2022a). Nevertheless, the precise attributes associated with m6A-related lncRNAs regarding prognostication and the immunological panorama of LSCC have not been extensively examined. Furthermore, there is lacking empirical proof pertaining to the influence of m6A-associated lncRNAs on the prognosis of LSCC individuals with a smoking background. Consequently, it is imperative to conduct additional investigations to elucidate these facets.

The purpose of this study was to analyze the key lncRNAs associated with m6A modifications in laryngeal samples obtained from patients and healthy donors, utilizing data derived from the TCGA database. Initially, we computed the expression levels of each lncRNA in every individual diagnosed with LSCC. Subsequently, a network analysis was conducted to investigate the relationship between m6A and lncRNAs. Our discoveries suggest that lncRNAs exhibit differential expression patterns between tumor and normal samples, and all m6A-associated lncRNAs can be categorized into two distinct groups based on cumulative distribution function or risk score. These categories demonstrate significant prognostic disparities, establishing the potential of the identified m6A-related lncRNAs in this analysis as prognostic biomarkers for predicting the outlook of LSCC. Additionally, through our investigation, we have identified immune cells with an association to risk, which could serve as diagnostic indicators for immunotherapy and promising targets for treating LSCC. Furthermore, we delve into a comprehensive examination of how each clinical phenotype impacts the prognosis of LSCC and analyze the corresponding survival curves. Ultimately, we identify one differentially expressed m6A-related lncRNA and one immune cell between LSCC patients with a history of smoking and those without.

The expression levels of m6A-related lncRNAs were examined in LSCC tumors and normal tissues. It has been established that these lncRNAs can impact the development of different types of cancers and can also regulate the expression of m6A regulators (Zhou et al., 2016), but how they interact with lncRNAs during LSCC progression is still unclear. At both transcriptional and post-transcriptional levels, gene expression and cellular biology are under the control of extensively modified lncRNAs by m6A regulators (Kopp and Mendell, 2018). In pancreatic cancer, IGF2BP2, a reader of m6A, enhances stem cell properties and carcinogenesis by stabilizing DANCR RNA and regulating the expression of lncRNA DANCR (Hu et al., 2020). Osteosarcoma tissues exhibit overexpression of SNHG12, which then leads to an increase in the tumorigenesis and metastasis induced by Notch2-and insulin-like growth factor 1 receptor (IGF1R) through the regulation of miR-195-5p (Zhou et al., 2018; Xu et al., 2020). Additionally, lncRNAs have the potential to function as competing endogenous RNA (ceRNA) that target m6A regulators and thereby influence tumor invasiveness (Li et al., 2022b). SNHG8, an m6A-related lncRNA, could stimulate the growth and migration of osteosarcoma cells by acting as a ceRNA to sponge miR-876-5p and miR-542-3p (Hao et al., 2020; Zhong et al., 2020). We believe that focusing on the interactions between m6A modifications and lncRNAs is crucial because lncRNA is a significant target of m6A modification regulators. By studying these interactions, researchers can identify potential prognostic markers or therapeutic targets for cancer, and may lead to significant advancements in cancer research and treatment.

In this investigation, it was observed that tumor samples exhibited notably higher expression levels of m6A-related lncRNAs in contrast to normal samples. However, the role of these lncRNAs in LSCC is not well understood. It has been reported that LINC02043, one of the identified lncRNAs, can be used as a predictor of recurrence-free survival in alcohol-related hepatocellular carcinoma. Higher expression of LINC02043 showed strong associated with a lower recurrence-free survival rate (Luo et al., 2020). In the context of LSCC, it is possible that high expression of LINC02043 indicates a higher risk and lower OS. Another identified lncRNA, STARD7-AS1, has been identified as a prognostic biomarker for autophagy-related lncRNA signaling in cervical cancer patients. Its elevation is considered a protective factor for cervical cancer (Feng et al., 2021). Nevertheless, the exact functions of these lncRNAs in LSCC are still unclear, and additional investigations are imperative to elucidate their roles. Additionally, some of the identified lncRNAs, such as AC011370.1 and AC090517.2, do not have a clear association with cancer, highlighting the need for more research to elucidate their significance in LSCC.

LINC00528, a gene that emerges as a key protecting player in tumor progression (Gong et al., 2020; Zhang et al., 2021), has demonstrated its potential in impeding cancer development by inducing programmed cell death and restraining cellular proliferation under laboratory conditions (Liu et al., 2020). In patients with HER-2 positive breast cancer, high expression levels of LINC00528 were associated with longer OS (Zhang et al., 2021). Interestingly, LINC00528 was found to be expressed at higher levels in smoking patients with LSCC, but it seems did not affect the prognosis or tumor progression between smoking and non-smoking patients. Therefore, the expression of LINC00528 was further analyzed in the normal group and in both smoking and non-smoking groups, along with its association with OS and PFS. Based on the findings, it was discovered that the group engaged in smoking displayed increased levels of LINC00528 expression. Furthermore, the heightened expression of LINC00528 was found to be linked with improved OS and PFS. In contrast, LSCC patients without a smoking history had a worse prognosis. Through analysis using the OncoPredict package, GSK591, ML323, PF-4708671, and Vorinostat were identified as potential sensitive drugs for treating LSCC patients with a smoking history. In addition, it was observed that there was a significant increase in Regulatory T cells (Tregs) infiltration in smoking patients. Because there are not survival differences between the smoke and the non-smoke group, Tregs may would not affect the survival rate whether patients have a smoke history. However, several studies showed that Tregs may affect the procession and prognosis of LSCC. Tregs is a unique subpopulation of CD4 T cells characterized by expression of the forkhead box P3 (FOXP3) transcription factor and high levels of CD25 (Hori et al., 2003; d’Hennezel and Piccirillo, 2011). Tregs play a major role in dampening spontaneous tumor-associated antigen (TAA)-specific immune responses (Sakaguchi, 2005; Zou, 2006). Moreover, radiotherapy can increase the recruitment of Tregs to the local TME and attenuate radiation-induced tumor death (Muroyama et al., 2017). Tregs were shown to be increased in the tumor and blood of HNSCC patients compared with healthy donors and their presence correlated with low CD8/Treg ratio (Lechner et al., 2017). Oweida et al. (2018) previously demonstrated that Tregs are highly enriched in orthotopic models of HNSCC and contribute to treatment resistance, also they found that STAT3 inhibition is a viable and potent therapeutic target against Tregs (Oweida et al., 2019). In conclusion, Treg is a worthwhile cell to influence the process and treatment effect of LSCC, but relationship between Treg and smoking is still unclear and worth deeper research.

Clusters based on m6A-related lncRNA classification exhibit distinct clinical outcomes and feature distributions, indicating their potential as representative molecular subpopulations. Additionally, these clusters are associated with different TME landscapes, suggesting a possible role of m6A-related lncRNAs in shaping the TME. Considering the clinical characteristics of LSCC patients, m6A-related lncRNAs have a significant impact, possibly mediated through alterations in the TME. The age of patients emerges as a crucial factor influencing patient survival in both cluster groups. Notably, cluster 1 patients display a poorer prognosis compared to cluster 2 patients, which could be attributed to higher levels of resting CD4^+^ T cells, NK cells, and naive B cells infiltrating the TME.

The risk model for LSCC, which relies on 15 m6A-related lncRNAs, efficiently segregates LSCC individuals into categories of high-risk and low-risk. In comparison to the low-risk group, the high-risk group exhibits a diminished OS rate. The risk model has a higher AUC value than conventional clinical parameters, suggesting good sensitivity and specificity. The expression patterns of m6A-related lncRNAs and diverse clinical characteristics exhibit notable variations among the two risk groups, encompassing age, gender, grades, and the levels of expression for N1-3, M0, T, III-VI, PD-L1. This indicates that our risk model not only holds clinical predictive ability but also offers insights for personalized treatment targeting methylation-related mechanisms. Our risk model for LSCC makes use of 15 lncRNAs related to m6A, effectively classifying patients with LSCC into groups with varying levels of risk. The low-risk group demonstrates a higher OS rate compared to the high-risk group, thus emphasizing the clinical significance of our proposed model. Furthermore, the risk model demonstrates better predictive ability compared to conventional clinical parameters, as evidenced by its higher AUC value. Importantly, the two risk groups differ significantly in terms of the expression of m6A-related lncRNAs and various clinical features, such as age, sex, grades, and expression levels of N1-3, M0, T, III-VI, PD-L1. This emphasizes the potential of our risk model in guiding personalized treatment strategies that target methylation-related mechanisms.

Our study discovered that the infiltration of macrophage M0 increased with higher risk scores. Several studies have reported that the infiltration of specific immune cells correlated with the prognosis of LSCC. It was reported that the favorable prognostic impact of higher tumor-infiltrating lymphocytes in patients with LSCC (Spector et al., 2019; Chatzopoulos et al., 2020). Chen et al. (2021) found that high infiltration of CD8 T cells, CD4 T cells, and M1 macrophages may correlated with more benefit from immune checkpoint inhibitor (ICI) therapy, and high infiltration of B cells, M0 macrophages, as well as M2 macrophages associated with less benefit from ICI therapy. By examining the expression of ICIs and modulating the expression of m6A regulators or lncRNAs, we can potentially alter the immune microenvironment and its associated biological processes (Xu et al., 2019; Zhang et al., 2020). In conclusion, our findings may potentially indicate macrophage M0 as a viable biomarker for prognosticating the overall survival of patients diagnosed with LSCC, as well as offer hope in individuals who may derive greater benefits from immunotherapeutic interventions.

However, our study does have limitations. First and foremost, it is important to note that only the LSCC cohort in TCGA was included in our analysis. We were unable to conduct an analysis of lncRNA due to the absence of clinical prognosis data in the microarray-based databases available in Gene Expression Omnibus (GEO). Therefore, extensive prospective studies and additional bioinformatic analyses will be necessary to validate the model’s accuracy. Furthermore, there are still many m6A-related lncRNAs associated with LSCC that remain unidentified, and the sensitivity of drugs also needs to be validated. To fully understand the functions and confirm the results, further bioinformatics analysis and experimental validation are essential. Lastly, the specific mechanisms through which our risk models predict prognosis are not yet clear and warrant further investigation.

In conclusion, this research analyzes the m6A-related lncRNAs found in the TCGA-LSCC database. The study presents a thorough and trustworthy framework for examining the potential of m6A-related lncRNAs in predicting the clinical features, prognosis, and TME of individuals diagnosed with LSCC. The present investigation additionally demonstrated the association involving LINC00528, smoking history, corresponding prognosis, and potential sensitive medications. Simultaneously, the alteration of LINC00528 was verified through the application of donor tissues from patients diagnosed with LSCC. These findings have the potential to assist in making clinical decisions and formulating personalized treatment plans for LSCC patients.

## Data Availability

The original contributions presented in the study are included in the article/Supplementary Material, further inquiries can be directed to the corresponding authors.
